# Plant Parasitic Nematode Identification in Complex Samples with Deep Learning

**DOI:** 10.2478/jofnem-2023-0045

**Published:** 2023-10-16

**Authors:** Sahil Agarwal, Zachary C. Curran, Guohao Yu, Shova Mishra, Anil Baniya, Mesfin Bogale, Kody Hughes, Oscar Salichs, Alina Zare, Zhe Jiang, Peter DiGennaro

**Affiliations:** Department of Electrical & Computer Engineering, University of Florida, Gainesville, Florida, 32611; Department of Computer & Information Science and Engineering, University of Florida, Gainesville, Florida 32611; Department of Entomology and Nematology, University of Florida, Gainesville, Florida 32611

**Keywords:** detection, diagnosis, identification, deep learning, method, technique

## Abstract

Plant parasitic nematodes are significant contributors to yield loss worldwide, causing devastating losses to every crop species, in every climate. Mitigating these losses requires swift and informed management strategies, centered on identification and quantification of field populations. Current plant parasitic nematode identification methods rely heavily on manual analyses of microscope images by a highly trained nematologist. This mode is not only expensive and time consuming, but often excludes the possibility of widely sharing and disseminating results to inform regional trends and potential emergent issues. This work presents a new public dataset containing annotated images of plant parasitic nematodes from heterologous soil extractions. This dataset serves to propagate new automated methodologies or speedier plant parasitic nematode identification using multiple deep learning object detection models and offers a path towards widely shared tools, results, and meta-analyses.

Accurate and efficient nematode identification is of paramount significance to define nematode diversity and implement effective control measures. Nematode diagnostic laboratories (NDL) play a critical role in this process by utilizing various techniques to detect, identify, and quantify nematode populations in samples. Plant-parasitic nematode (PPN) accounts for over 10% of the annual life-sustaining crop losses, costing the industry roughly 100–157 billion U.S. dollars per year (Chitwood 2003; Abad et al., 2008). In some cropping systems, PPN are the dominant plant-pathogens of any kind (Savary et al., 2019). Thus, NDL receive a large number of samples (Zasada et al., 2019) from farmers and agricultural consultants for the diagnosis and identification of PPN present in their fields. This information is critical for developing effective management strategies to control the nematode population, spread of nematodes, and minimize economic losses. Early detection of emerging nematode species is especially important, as it allows for rapid implementation of control measures to prevent the spread of those species. Since NDL receive a large number of samples every year, the manual effort required for nematode identification can result in long turnaround times (Patrick, 2022), delaying control strategy implementation and potentially exacerbating the economic impact of nematode damage on crops. Therefore, the integration of machine learning approaches in NDL identification methods has the potential to speed up the identification process, reducing turnaround times and providing more accurate and efficient diagnosis of nematode populations.

Current nematode identification involves morphological and molecular detection methods (Ye et al., 2019; Cunha et al., 2018), which are highly accurate but have limitations such as the need for microscopy equipment, highly trained nematology technicians, and turnaround times as long as 15 weeks (Patrick, 2022), especially for large numbers of samples, making it necessary to find methods that can identify nematodes quickly in order to determine the appropriate treatment. Identifying and quantifying the abundance of PPN species is crucial, as their damage threshold levels vary from 10 to 500 per 100 cc of soil (Barker et al., 1985). This process requires a varying amount of time and a trained nematology taxonomist to ensure the correct species identification. In addition, the presence of a dominant nematode species in a sample can create unconscious bias in human image analysis, potentially hindering the early detection of emerging species. This is particularly relevant in the context of quarantine issues, where the timely identification of invasive or new nematode species is critical for preventing their spread to other areas. The inclusion of machine learning approaches in nematode identification has the potential to address this issue by reducing the reliance on human image analysis and providing more accurate species identification, including the identification of emerging species. This could help improve the effectiveness of quarantine measures by facilitating the early detection and rapid response to invasive nematode genera.

Artificial intelligence, sometimes referred to as deep learning, utilizes deep neural networks for image segmentation, object detection and classification (Zare et al., 2018). Specifically, object detection models are able to identify and quantify nematodes from a single image. This technique is especially suitable for handling large number of samples as well as detecting unexpected nematodes in routine samples and resolving plant parasitic nematodes in complex backgrounds. In recent years, deep learning has successfully been used in plant pest classification and detection (Cheng et al., 2017; Xie et al., 2018) with a level of accuracy of up to 93% (Kasinathan et al., 2021; Picek et al., 2022). This approach has been promising in shortening the fungus classification and identification process by two to three days, reducing the cost of diagnosis (Zielinski et al, 2020). For nematodes, there has been significant improvement in the quantification time of soyabean cyst nematode eggs while maintaining human-level accuracy and avoiding inter-rate and intra-rate variabilities (Akintayo et al., 2018), illustrating the remarkable promise of applying machine learning approaches to phenotyping for pest assessment and management. As thousands of nematode samples can be sent to a single NDL annually, there is a rich data resource for the training of deep neural networks. Briefly, nematode samples retained by the NDL can be imaged and annotated for specific nematode features by a nematode identification specialist, providing rich training labels for neural networks. The pretrained model is then used to automatically identify and classify nematodes according to their importance to agriculture and natural resources. Several models have already been developed and evaluated for classifying PPN at genus level using single nematode images (Sabrina et al 2022) with a high level of accuracy.

Here, we utilized nematode samples from the University of Florida Nematode Diagnostic Lab (UF NDL) to develop machine learning algorithms and evaluate different models. The pre-trained models were used to detect and quantify plant parasitic nematodes recovered from field soil using a common nematode extraction method with a high level of accuracy and precision. These findings indicate the potential of machine learning in improving the identification and quantification of plant parasitic nematodes, which can have significant implications for effective nematode management in agriculture.

## Materials and Methods

### Sample imaging and annotation

**Figure 1:**
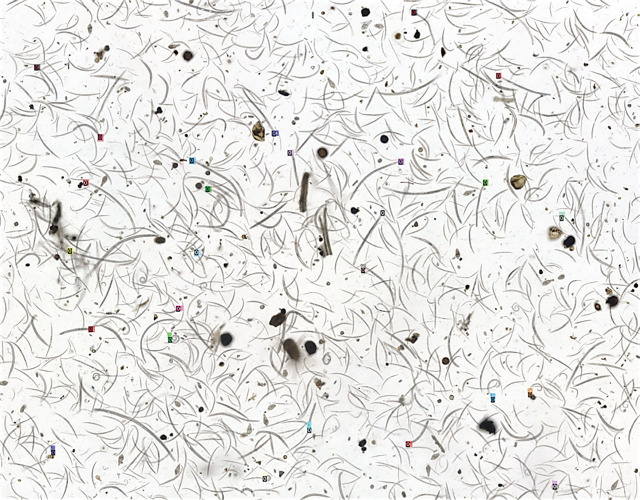
Representative sample image of nematodes extracted from soil samples sent to NDL. Colored boxes indicate annotations of plant parasitic nematode heads.

Nematode samples for imaging were received from UF NDL. Briefly, UF NDL received soil samples from growers to identify and quantify PPN in their field. Nematodes were extracted using sugar floatation and centrifugation method (Gooris and d’Hedre, 1972) using 250 cc of soil. Before imaging, all nematodes were concentrated, briefly heat killed, and placed in a 6 cm diameter Petri plate. Nematode samples were imaged using Keyence digital microscope BZ-X at 20X magnification with Z-stacking function. Z-stacking combines multiple images taken at different focal distances to provide a composite image with a greater depth of field (Johnson 2008; Ray 2002). About 5,000–10,000 images were stitched together to create each composite image, and a total of 100 composite images were collected. Samples encompass multiple hosts and locations, ensuring a range of PPN in each image. A representative image is shown in Figure 1, with colored boxes representing annotations of PPN.

The acquired composite images were imported to Visual Geometric Group (VGG) image annotator (Dutta, n.d.; annotate.officialstatistics.org). Each image was annotated for all potential plant parasitic nematode heads present in each image by drawing a box around a stylet. Each image contained between 10 and 100 stylet bearing nematodes.

The original resolution of collected images averaged 4,000 × 4,000 pixels. These raw, high-resolution images are not suitable for training CNN-based object detection models due to the large amount of memory needed. To alleviate this issue, a grid cropping strategy was implemented, splitting the original images via a sliding window of size 640 × 640 pixels. Each original image was split into roughly 39 sub-images using the sliding window grid-crop, resulting in 3,503 total crops from the 90 original images.

### Data Augmentation

Data augmentation was used to increase the number of labeled examples for model training, as well as to introduce more variety into the data. Specifically, within the models described this paper, we used translation, rotation, scaling, shearing, color space adjustments, and mosaic augmentations. For translation, each image was translated by a random proportion sampled from a normal distribution with mean 0.1; for rotation, a mean of 0.0 degrees; for scaling, a mean gain of 0.5; for shearing, a mean of 0.0 degrees. For the color space augmentations, the hue, saturation, and value were augmented. The mosaic augmentation samples from the above augmentations and combines four random images into one mosaic image (Wei et al., 2020).

### Model Training

The initial model training stage utilized various popular CNN-based object detection models to determine the feasibility of using these models for the detection task. Namely, CenterNet (Duan et al., 2019), EfficientDet (Tan et al., 2019) and RetinaNet (Lin et al., 2018), and YOLOv5 (Liu et al., 2022) were used to gather initial performance results and select the highest performing model for further experimentation. For the models, the size of the training dataset was increased by a ratio of 4:1 through the data augmentation pipeline described above. Each model was then trained on the data for 1,000 epochs, or until there was no significant change in the loss function. From this the best performing model was selected for further experimentation.

The performance of the detection task was evaluated using the following popular object detection evaluation metrics: mean average precision (mAP) at 0.5 intersection over union (IOU). MAP at 0.5 IOU is the average precision of the predicted bounding boxes where the degree of overlap between the detected bounding boxes and the ground truth bounding boxes is at least 50%. Precision is calculated as the number of true positives divided by the number of true positives and false positives. IOU is calculated by dividing the overlap between the predicted and ground-truth box by the combined area of both the predicted and ground-truth box. For nematode identification, this means that at least half (0.5) of the models predicted box overlaps with the annotated box drawn around the nematode stylet. A higher mAP means a greater number of predicted annotations overlapped by at least 0.5 with the actual annotation.

The baseline YOLOv5 model conducts both region detection and object classification simultaneously. YOLOv5 accomplishes this by first subdividing the input image into an s × s grid. Each box in the grid predicts the probabilities that the object covered in the box belongs to a given class. From these probabilities and boxes, a bounding box is then constructed around the object. The main components of YOLOv5 are the backbone, the neck, and the head. The “backbone” is responsible for extracting features from the image itself, while the “neck” performs feature fusion, the fusing of features from different levels in the backbone hierarchy. Finally, the “head” performs the actual predictions of bounding boxes and classes over the Fused features, which are mapped back to the original image. Similar approaches were used for the other object detection models, CenterNet (Duan et al., 2019), EfficientDet (Tan et al., 2019) and RetinaNet (Lin et al., 2018).

### Model improvements

After running the initial experiments, it was found that the baseline YOLOv5 model performed the best in terms of mean average precision. From this conclusion several architectural modifications were made to the YOLOv5 model to increase the accuracy of the model. This includes modifying the “neck,” “backbone,” and depth of the YOLOv5 model. The modified models were trained in the same manner as before, with 1,000 epochs of training preformed.

### BI-FPN-modified YOLOv5 model

The Bi-FPN variation of the YOLOv5 model tested in this work involves modifying the neck of the YOLO architecture to allow more flow between features of different levels. The intuition behind its inclusion in our YOLOv5 experiments comes from the idea that allowing a freer fusion of features will better allow the network to place emphasis on lower-level features which may be more important in identifying the small stylets of PPN. The efficacy of the BI-FPN neck in small object detection when used in conjunction with YOLO has also been seen in previous research, where it was shown that a bi-FPN neck outperformed other architectures in small object detection (Benjumea et al., 2021).

### DenseNet backbone-modified YOLOv5 model

The DenseNet variation of the YOLOv5 model involves the modification of the backbone of the YOLO architecture. The default backbone of YOLOv5 was replaced with a DenseNet backbone. DenseNet itself is an image classification CNN architecture (Huang et al., 2017). This work tested the DenseNet backbone as it has been found to be very effective in classifying nematodes (Abade et al., 2021), performing above other classification networks. This led to the hypothesis that DenseNet may better be able to identify useful features within the images required for nematode detection.

### Deeper-modified Yolov5 model

To achieve the deeper YOLOv5x model, the YOLO algorithm was simply made larger and deeper by a factor of two. This means that each layer's neuron count was doubled, and the number of repeated layers was also doubled in the backbone and neck of the model. Considering that the larger models showed better results within the released family of YOLOv5 models (YOLOv5n, YOLOv5s, YOLOv5m, YOLOv5l, and YOLOv5x), this work hypothesized that a larger, deeper model may yet further improve the performance of the algorithm in nematode detection.

## Results

### Model evaluation

Four different CNN object detection models were evaluated using the manually annotated stylets as input (Table 1). YOLOv5 performed the best, with a maximum mAP at 0.5 IOU of 0.787. This means that approximately 78% of the predicted objects overlapped with at least half of the manually annotated stylets. The other CNN object detection models performed similarly, but with slightly lower mAP scores corresponding to approximately 64–69% predicted objects overlapping with at least half of the manually annotated stylets (Table 1).

**Table 1.** Model and corresponding maximum mAP.

**Model**	**Max MAP_0.5**
YOLOv5x Baseline	0.787
CenterNet HourGlass104 512×512	0.6929
EfficientDet D1 640×640	0.6489
SSD ResNet50 V1 FPN 640×640	0.6439

### Modified YOLOv5

As the CNN object detection model YOLOv5 performed the best of all tested models, we sought to improve the model by modifying its architecture to increase efficacy in detecting nematode stylets indictive of potential plant parasitic nematodes. From the modified architectures, the best overall performance was seen in the YOLOv5x Bi-FPN model, with a validation mAP score at 0.5 IOU of 0.791. This means that approximately 79% of predicted objects overlapped with at least half of the manual annotations (Table 2). We also examined the maximum precision and maximum recall for modifications to the YOLOv5 model. Here, precision is defined as how often the model correctly predicts objects, indicating how much we can rely on the model's positive predictions; and recall defines as a measure of predicting all possible objects, indicating potential predictions the model may have missed. Together, these can inform the potential for false positive and false negative errors in the model's object detection. The best modification was seen with the Bi-FPN variation of the YOLOv5 model, yielding a maximum precision of approximately 85% and recall of 75% (Table 2). A higher precision compared to recall value indicates that the model is accurate when classifying a nematode stylet, but it may not classify all potential nematode stylets in a sample. Regardless, all models and model variations tested seem to produce relatively similar performance with no large gain being seen from the modifications applied (Table 2).

**Table 2.** Modified YOLOv5 models with precision and recall.

**Model**	**Max MAP_0.5**	**Max Precision**	**Max Recall**
YOLOv5x Baseline	0.787	0.836	0.772
YOLOv5x-Bi-FPN	0.791	0.851	0.753
YOLO-v5x Densenet	0.769	0.831	0.782
YOLOv5x-Deeper	0.779	0.831	0.774

## Discussion

Nematode population surveys are essential to understanding the occurrence, abundance, and diversity of PPN in an agricultural system. Such critical information is used to assess when a population increases above a damaging threshold, which leads recommendations for effective and economically viable nematode management strategies. Nematode surveys are also essential in preventing the introduction of quarantined pests, and in monitoring the temporal and biogeographical trends in plant diseases induced by plant-parasitic nematodes. Regional or local NDL are valuable resources utilized by growers, extensionists, and other stakeholders perform these surveys. Growers rely on NDL assessments to decide what to plant (regular crops, fallow crops, nematode traps, etc.), when to plant, and what desirable management approaches may be needed. In the Pacific Northwest of the United States, five NDL surveyed from 2012 to 2016 showed common PPN genera and distributions (Zasada, 2019). Similarly, a survey in Idaho between 2000 and 2006 reported prominent PPN genera (Hafez, 2010). The UF NDL regularly receives thousand of samples each year, with an incredible array of PPN diversity. However, different NDL may have different values for damage thresholds (the number of PPN representing significant damage to a specific crop) in a sample. These differences are likely due to regional specificities like climate, soil type, and cropping systems. For instance, Kansas has employed a threshold for the dagger nematode (Xiphinema spp.) at 150–200 nematodes per 100 cc soil (Todd and Jardine, 1993), while Iowa uses a threshold of 30–40 dagger nematodes per 100 cc of soil (Tylka, 2009). As climate, plant resistance, and integrated management practices change, coordination across different regions can help guide threshold levels and better direct nematode management. Due to the high volume and specificity of sample data collected by NDL, many are not part of the National Plant Disease Network (NDPN), which works to coordinate national pest management efforts. The goal of this work was to provide a platform and process of digitizing NDL sampling and record keeping to inform nematode management on a national level.

A major distinction of this work related to previous attempts at leveraging machine learning with nematode identification is the use of heterologous samples. These samples contain PPN, as well as free-living, predatory, and other non-plant parasitic nematodes. Our images represent the real-world challenges of identifying and counting PPN from field samples. This does pose some issues however, specifically the potential for false positive and false negative results. While all models performed similarly, the best performer was the Bi-FPN variation of the YOLOv5 (Table 2). With a mAP at 0.5 IOU of approximately 80% and using the Iowa State threshold values for common PPN, in practice this model would successfully place most samples correctly within the ‘above’ or ‘below’ damage threshold categories. This is due to the variability allowed within damage thresholds (30–40 dagger nematode, 500–1,000 spiral nematodes per 100 cc of soil, etc.). Some nematode genera, however, have very low damage thresholds, such that even one nematode found in 100 cc of soil is cause for concern (e.g., sting nematode; Tylka, 2009). PPN numbers alone are not the sole basis for recommendations. Cropping history, time of year, and soil type also play important roles. This “meta” data is currently handled by experts but, to the best of our knowledge, not used at a national or regional level to inform changes in trends of nematode virulence or population movements. Digitizing this wealth of “meta” data and datasets produced from NDL would provide significant opportunities to forecast nematode pressures and synchronize management efforts.

We examined each model's precision and recall as the variation seen within PPN damage thresholds values the importance of correct identification over correct counting. The Bi-FPN variation of the YOLOv5 model had a slightly higher maximum precision than maximum recall (Table 2), indicating a higher chance that the model will correctly call a PPN rather than calling all PPN in a sample. Most false positives were called on nematode heads that did not contain a stylet but displayed darker pharyngeal patterns. Most false negatives were missed on nematodes that had poorly defined stylets due to microscope image artifacts like debris or poor lighting. The next step will be to integrate these models into previously developed models that focused on the identification of specific nematode genera. The models presented in this paper delineate potential PPN in a heterologous sample, while other efforts have targeted specific nematode genera in “clean” homogenous samples (Akintayo et al., 2018; Uhlemann et al., 2020; Qing et al., 2022; Shabrina et al., 2023).

Another benefit to digitizing NDL to nationally coordinate nematode diagnostics is to combat emerging nematode pathogens. There are two current issues with detecting new PPN genera or species in a region: 1) regional expertise may be limited and unable to readily detect uncommon and new species invasions on routine samples, and 2) due to national quarantine, soil samples are unable to cross some state lines, limiting expert exposure to samples from certain areas of the country. Employing machine learning algorithms to augment NDL diagnostics can mediate both of these significant issues as all PPN and genera can be detected objectively, and microscopic images are not under quarantine. The wide use of machine learning in nematode diagnostics can also increase the precision and recall of these models over time.

The goal of this work was to demonstrate the utility of machine learning in nematode diagnostics. The models presented distinguish stylet-bearing nematodes from non-stylet-bearing nematodes, and while this does not necessarily delineate plant parasitism alone, in front of other developed models for identifying specific genera, we hold this is a powerful tool that can revolutionize PPN management.

## References

[j_jofnem-2023-0045_ref_001] Abad P., Gouzy J., Aury J.M., Castagnone-Sereno P., Danchin E.G., Deleury E., Perfus-Barbeoch L., Anthouard V., Artiguenave F., Blok V.C., Caillaud M.C. (2008). Genome sequence of the metazoan plant-parasitic nematode *Meloidogyne incognita*. Nature biotechnology.

[j_jofnem-2023-0045_ref_002] Abade A. D. S., Porto L. F., Ferreira P. A., Vidal F. D. B. (2021). Nemanet: A convolutional neural network model for identification of nematodes soybean crop in Brazil. arXiv preprint.

[j_jofnem-2023-0045_ref_003] Akintayo A., Tylka G. L., Singh A. K., Ganapathysubramanian B., Singh A., Sarkar S. (2018). A deep learning framework to discern and count microscopic nematode eggs. Scientific reports.

[j_jofnem-2023-0045_ref_004] Barker K. R., Schmitt D. P., Imbriani J. L. (1985). Nematode population dynamics with emphasis on determining damage potential to crops. An advanced treatise on Meloidogyne.

[j_jofnem-2023-0045_ref_005] Benjumea A., Teeti I., Cuzzolin F., Bradley A. (2021). YOLO-Z: Improving small object detection in YOLOv5 for autonomous vehicles. arXiv preprint.

[j_jofnem-2023-0045_ref_006] Cheng X., Zhang Y., Chen Y., Wu Y., Yue Y. (2017). Pest identification via deep residual learning in complex background. Computers and Electronics in Agriculture.

[j_jofnem-2023-0045_ref_007] Chitwood D.J. (2003). Research on plant-parasitic nematode biology conducted by the United States Department of Agriculture–Agricultural Research Service. Pest Management Science: Formerly Pesticide Science.

[j_jofnem-2023-0045_ref_008] Duan K., Bai S., Xie L., Qi H., Huang Q., Tian Q. (2019). Centernet: Keypoint triplets for object detection.

[j_jofnem-2023-0045_ref_009] Dutta A. VGG Image Annotator.

[j_jofnem-2023-0045_ref_010] Gooris J., d’Herde C. J. (1972). A method for the quantitative extraction of eggs and second stage juveniles of Meloidogyne spp. from soil.

[j_jofnem-2023-0045_ref_011] Huang G., Liu Z., Van Der Maaten L., Weinberger K. Q. (2017). Densely connected convolutional networks.

[j_jofnem-2023-0045_ref_012] Johnson D. (2008). How to Do Everything: Digital Camera [Online].

[j_jofnem-2023-0045_ref_013] Kasinathan T., Singaraju D., Uyyala S. R. (2021). Insect classification and detection in field crops using modern machine learning techniques. Information Processing in Agriculture.

[j_jofnem-2023-0045_ref_014] Kranse O.P., Ko I., Healey R. (2022). A low-cost and open-source solution to automate imaging and analysis of cyst nematode infection assays for *Arabidopsis thaliana*. Plant Methods.

[j_jofnem-2023-0045_ref_015] Lin T. Y., Goyal P., Girshick R., He K., Dollár P. (2017). Focal loss for dense object detection.

[j_jofnem-2023-0045_ref_016] Liu H., Sun F., Gu J., Deng L. (2022). Sf-yolov5: A lightweight small object detection algorithm based on improved feature fusion mode. Sensors.

[j_jofnem-2023-0045_ref_017] Patrick (2022). Turnaround times for routine nematode testing expected to be long.

[j_jofnem-2023-0045_ref_018] Picek L., Šulc M., Matas J., Heilmann-Clausen J., Jeppesen T. S., Lind E. (2022). Automatic fungi recognition: Deep learning meets mycology. Sensors.

[j_jofnem-2023-0045_ref_019] Ray S. F. (2002). Applied Photographic Optics.

[j_jofnem-2023-0045_ref_020] Qing X., Wang Y., Lu X., Li H., Wang X., Li H., Xie X. (2022). NemaRec: A deep learning-based web application for nematode image identification and ecological indices calculation. European Journal of Soil Biology.

[j_jofnem-2023-0045_ref_021] Shabrina N. H., Lika R. A., Indarti S. (2023). Deep learning models for automatic identification of plant-parasitic nematode. Artificial Intelligence in Agriculture.

[j_jofnem-2023-0045_ref_022] Tan M., Pang R., Le Q. V. (2019). Efficientdet: scalable and efficient object detection. arXiv. arXiv preprint.

[j_jofnem-2023-0045_ref_023] Todd T. C., Jardine D. J. (1993). Nematodes: management guidelines for Kansas crops.

[j_jofnem-2023-0045_ref_024] Uhlemann J., Cawley O., Kakouli-Duarte T. (2020). Nematode Identification using Artificial Neural Networks.

[j_jofnem-2023-0045_ref_025] Wei Z., Duan C., Song X., Tian Y., Wang H. (2020). Amrnet: Chips augmentation in aerial images object detection. arXiv preprint.

[j_jofnem-2023-0045_ref_026] Xie C., Wang R., Zhang J., Chen P., Dong W., Li R., Chen T., Chen H. (2018). Multi-level learning features for automatic classification of field crop pests. Comput Electron Agric.

[j_jofnem-2023-0045_ref_027] Zasada I.A., Kitner M., Wram C., Wade N., Ingham R.E., Hafez S., Mojtahedi H., Chavoshi S., Hammack N. (2019). Trends in occurrence, distribution, and population densities of plant-parasitic nematodes in the Pacific Northwest of the United States from 2012 to 2016. Plant Health Progress.

[j_jofnem-2023-0045_ref_028] Zieliński B., Sroka-Oleksiak A., Rymarczyk D., Piekarczyk A., Brzychczy-Włoch M. (2020). Deep learning approach to describe and classify fungi microscopic images. PLoS ONE.

